# The impact of psychological aspects, age, and BMI on eating disorder psychopathology among adult males and females with type 1 diabetes

**DOI:** 10.1177/2055102920975969

**Published:** 2020-11-24

**Authors:** Line Wisting, Cecilie Siegwarth, Torild Skrivarhaug, Knut Dahl-Jørgensen, Øyvind Rø

**Affiliations:** 1 Regional Department for Eating Disorders, Division of Mental Health and Addiction, Oslo University Hospital, Norway; 2 Oslo Diabetes Research Centre, Oslo, Norway; 3 The Norwegian Diabetes Centre, Oslo, Norway; 4 Division of Childhood and Adolescent Medicine, University of Oslo, Norway; 5 Institute of Clinical Medicine, Faculty of Medicine, University of Oslo, Norway; 6 Institute of Clinical Medicine, Mental Health and Addiction, University of Oslo, Norway

**Keywords:** eating disorders, health psychology, illness perceptions, psychological correlates, type 1 diabetes

## Abstract

This study investigated correlates of eating disorder (ED) psychopathology among adults with type 1 diabetes (T1D). A total of 282 males (*n* = 112) and females (*n* = 170) with T1D (18–79 years) participated. Overall, psychological aspects (i.e. illness perceptions, coping strategies, insulin beliefs, anxiety, and depression) were associated with ED psychopathology. Associations were generally stronger among females than males. In a regression model, age, BMI, personal control, and anxiety explained 51% of the variance in ED psychopathology among females, whereas BMI, personal control, and anxiety explained 47% of the variance among males. Greater clinical awareness of health psychological aspects may contribute to reduce the risk of developing ED.

## Introduction

Type 1 diabetes (T1D) is a chronic autoimmune disease characterized by lack of insulin production in the pancreas, leading to elevated blood glucose levels and a lifelong need to administer insulin to regulate blood glucose levels and prevent increased risk of morbidity and mortality (Gagnum et al., 2015, 2017; International Diabetes Federation, 2006). Eating Disorders (ED) are characterized by disordered eating behaviors (e.g. dietary restriction, binge eating, and purging), distorted body image, and intrusive thoughts and concerns about body weight and shape (American Psychiatric Association, 2013).

T1D is associated with a higher risk for developing eating disorders (ED), with a 2–3 fold increased prevalence of ED in females with T1D compared to females without T1D (Mannucci et al., 2005; Nielsen, 2002; Young et al., 2012). Intentional insulin omission for weight control is reported in up to 30% of females with T1D (Goebel-Fabbri et al., 2008). T1D and comorbid ED are associated with increased risk of diabetes complications and increased rates of mortality (Nielsen et al., 2002), underscoring the importance of knowledge about potential predictors for the development of ED in this patient group.

The etiology of ED is generally recognized as multifactorial and complex, including genetic, environmental, and cultural aspects (Striegel-Moore and Bulik, 2007). Specifically, female sex and late adolescent/early adult age represent a high-risk period for the development of ED, which has also been supported among individuals with T1D (Colton et al., 2015). Reported correlates of ED psychopathology in adults with T1D include negative affect (Merwin et al., 2015; Moskovich et al., 2019), poorer executive functioning (Broadley et al., 2018), social media usage (Verbist and Condon, 2019), body image dissatisfaction (Verbist and Condon, 2019), depression (Olmsted et al., 2008), and anxiety (Bernstein et al., 2013). Psychological aspects such as coping strategies, insulin beliefs, and illness perceptions have been found to be associated with ED psychopathology among adolescent females with T1D, but not among adolescent males (Wisting et al., 2015). However, knowledge is lacking as to how these variables interact with ED psychopathology among adult males and females with T1D. Although it is commonly reported that mean levels of ED are higher in females than in males also among adults with T1D, it is unknown whether the gender differences in *associations* between psychological variables and ED psychopathology observed among adolescents are also evident among adults with T1D. Such knowledge may have important clinical implications and potentially contribute to better identify individuals at risk of developing ED, and target prevention- and treatment efforts for this at-risk group. Most studies on diabetes and eating disorders focus on adolescent and young adult females, and knowledge is scarce on males (all age groups) and older adult females (i.e. >40 years) (Young et al., 2012).

This study therefore aimed to investigate the impact of psychological aspects (i.e. illness perceptions, coping strategies, insulin beliefs, depression, and anxiety), age, and BMI, on ED psychopathology among adults with T1D, with a particular focus on gender differences. Specifically, we hypothesized that psychological aspects in general, and personal control in particular, would be more strongly associated with ED psychopathology among females than males.

## Material and methods

### Design

This is a cross-sectional study of disturbed eating and diabetes psychology among adults with T1D, recruited from the Norwegian Diabetes Centre, Oslo, Norway.

### Participants and procedure

Methodologies are described in details in previous studies (Wisting et al., 2018, 2019a), and are therefore summarized more briefly in the current study. In this observational diabetes psychology study, a total of 282 males (40%) and females (60%) with T1D aged 18–79 years participated in the study (mean age 42.11; SD: 15.19). Sample characteristics are presented in Table 1. The regional ethics committee approved the study, and written consent was obtained from all participants.

**Table 1.** Participant characteristics.

	All	Males	Females	Sig. level	Effect size
	*N* = 282	*N* = 112 (40%)	*N* = 170 (60%)		
Age (years)	42.11 (15.19)	44.57 (15.92)	40.47 (14.49)	0.05	0.27
Diabetes onset (age)	15.14 (11.18)	15.43 (10.92)	14.94 (11.38)	ns	
HbA1c % (mmol/mol)	7.75 (61.2) (0.91)	7.61 (59.7) (0.89)	7.85 (62.3) (0.91)	ns	
Diabetes duration (years)	27.09 (14.44)	29.14 (14.82)	25.71 (14.05)	ns	
BMI (kg/m²)	25.96 (4.13)	26.47 (3.82)	25.63 (4.30)	ns	
Mode of insulin treatment	56.3% pen	60.9% pen	53.4% pen		
	43.3% pump	38.0% pump	46.6% pump		
DEPS-R total	13.83 (9.16)	11.18 (7.80)	15.57 (9.59)	0.001	−0.50
HAD depression	3.75 (3.61)	3.53 (3.35)	3.90 (3.77)	ns	
HAD anxiety	6.39 (4.27)	5.12 (3.67)	7.26 (4.44)	0.001	−0.53

Data are mean (SD). Significance level (*p* < 0.001, 0.01, and 0.05) and effect size (ES) estimation (Cohen’s *d*) is calculated when differences are significant.

ns: not statistical significant differences.

### Measures

The Diabetes Eating Problem Survey-Revised (DEPS-R) (Markowitz et al., 2010) is a diabetes-specific screening tool for disturbed eating and consists of 16 items (e.g. *I feel fat when I take all of my insulin*). Responses range from 0 to 6 (from *never* to *always*), and higher scores indicate greater ED pathology. Scores of 20 or more indicates a level of disturbed eating warranting further attention. The DEPS-R has been translated and validated among Norwegian adolescents (Wisting et al., 2013) and adults (Wisting et al., 2019b).

The Brief Illness Perception Questionnaire (BIPQ) (Broadbent et al., 2006) measures illness perceptions, including *personal control* (*How much control do you feel you have over your diabetes*), *treatment control, identity, coherence, emotional representations*, and *concern* (Broadbent et al., 2006). Answers range from 0 to 10, and higher scores indicate more negative views of their T1D.

The Beliefs about Medicines Questionnaire (BMQ) (Horne and Weinman, 1999) has been translated and validated in Norwegian (Jonsdottir et al., 2009) and the specific concern subscale was utilized in the current study to measure insulin concern (e.g. *Having to take insulin concerns me)*. Answers range from 1 (*strongly disagree*) to 5 (*strongly agree*).

The COPE Inventory (Carver et al., 1989) measures various coping responses when confronted with stressful events (e.g. *I try to get emotional support from friends or relatives*), and answers range from 1 (*I usually don’t do this at all*) to 4 (*I usually do this a lot*). The current study included the subscales *focus on and venting of emotions, active coping, use of emotional social support*, and *denial*.

The Hospital Anxiety and Depression Scale (HADS) (Zigmond and Snaith, 1983) utilized the subscales for anxiety and depression. The HADS consists of 14 items (e.g. *I feel restless as I have to be on the move*) and answers range from 0 to 3, with higher scores indicating higher severity. The Norwegian version of HADS has previously demonstrated satisfactory psychometric properties (Leiknes et al., 2016).

Clinical data was assessed via the Norwegian Quality Improvement of Laboratory Examinations (NOKLUS) system. BMI was calculated based on self-reported weight and height (kg/m²).

### Data analysis

Pearson correlations were conducted to investigate associations between variables. Subsequent to the correlation analyses, standard multiple regression (enter) analyses was conducted with significant correlations (*p* < 0.05) in line with the backward elimination strategy described below, to investigate possible risk factors for disturbed eating. Analyses were split by gender to examine gender differences. Statistical analyses were conducted using SPSS (IBM Corp, 2015).

## Results

### Participant characteristics

Table 1 illustrates sample characteristics. Age range was 18–79 years, mean age 42.11 (SD: 15.19), mean Hemoglobin A1c (HbA1c as a measure of blood glucose control) was 7.75% (61.2 mmol/mol) (SD: 0.91), and mean BMI was 25.96 (SD: 4.13). All patients used modern, intensified insulin treatment: A total of 56.3% used a basal-bolus regimen with >4 injections a day with insulin pens. 43.3% used insulin pumps. With regard to psychopathology, mean DEPS-R score was 13.83 (9.16) for the total population, 11.18 (7.80) for males, and 15.57 (9.59) for females. As reported in a previous study (Wisting et al., 2018), 13.3% of the males and 24.8% of the females scored above the cut-off for disturbed eating on the DEPS-R, and mean DEPS-R total score was significantly associated with HbA1c among females (0.27, *p* < 0.01), but not among males.

### Associations with eating disorder psychopathology

Table 2 shows that among females, ED psychopathology (DEPS-R total score) was significantly associated with age (−0.32, *p* < 0.001), BMI (0.33, *p* < 0.001), anxiety (0.48, *p* < 0.001), and depression (0.47, *p* < 0.001). Furthermore, ED psychopathology was significantly correlated with all BIPQ dimensions (correlation coefficients ranging from 0.28 to 0.57, all *p’s* < 0.001), the COPE scales venting emotions and denial (0.23, *p* < 0.01 and 0.30, *p* < 0.001, respectively), as well as BMQ insulin concern (0.32, *p* < 0.001). Among males, ED psychopathology was significantly associated with BMI (0.35, *p* < 0.001), depression (0.39, *p* < 0.001), and anxiety (0.50, *p* < 0.001), as well as all illness perception dimensions (correlation coefficients ranging from 0.20 to 0.51, *p’s* ranging from <0.05 to 0.001), and active coping (−0.24, *p* < 0.05).

**Table 2.** Correlations between age, BMI, psychological aspects, and ED psychopathology among males and females with T1D.

	ED psychopathology	
	Males	Females
Age	ns	−0.32***
BMI	0.35***	0.33***
BIPQ consequences	0.29**	0.41***
BIPQ personal control	0.51***	0.57***
BIPQ treatment control	0.20*	0.30***
BIPQ identity	0.22*	0.28***
BIPQ coherence	0.33**	0.30***
BIPQ emotional representations	0.30**	0.49***
BIPQ concern	0.22*	0.45***
BMQ insulin concern	ns	0.32***
COPE venting emotions	ns	0.23**
COPE active coping	−0.24*	ns
COPE emotional social support	ns	ns
COPE denial	ns	0.30***
HAD depression	0.39***	0.47***
HAD anxiety	0.50***	0.48***

Data are correlation coefficients (Pearson).

Significance level: ***p < 0.001; **p < 0.01; *p < 0.05.

### Explained variance in eating disorder psychopathology

As described in the data analysis section, significant correlations (Table 2) were entered into a regression model (Table 3). Among females, the BIPQ dimensions consequences, personal control, treatment control, identity, coherence, emotional representations, and concern, in addition to BMQ insulin concern, age, BMI, HAD anxiety and depression, and the COPE subscales venting emotions and denial, were entered into the equation. This model explained 56% of the variance in the DEPS-R total score. After removing non-significant variables one by one in line with the backward elimination strategy, age (β −0.14, *p* < 0.05), BMI (β 0.26, *p* < 0.001), personal control (β 0.42, *p* < 0.001), and anxiety (β 0.29, *p* < 0.001) remained significant, explaining 51% of the variance in ED psychopathology (Table 3). Among males, BMI, all BIPQ dimensions, in addition to COPE active coping and HAD depression and anxiety, were entered into the regression equation. This model explained 53% of the variance in the DEPS-R total score. BMI (β 0.31, *p* < 0.001), personal control (β 0.33, *p* < 0.001) and anxiety (β 0.40, *p* < 0.001) remained significant subsequent to the backward elimination strategy, explaining 47% of the variance in ED psychopathology.

**Table 3.** Regression model with ED psychopathology as the dependent variable, and age, BMI, and psychological aspects as independent variables.

Females	B	Std. Error	Beta (β)	*t*	Sig.
Age	−0.09	0.04	−0.14	−2.32	0.05
BMI	0.57	0.13	0.26	4.42	0.001
BIPQ personal control	1.94	0.29	0.42	6.71	0.001
HAD anxiety	0.63	0.13	0.29	4.68	0.001
Males	B	Std. Error	Beta (β)	*t*	Sig.
BMI	0.70	0.17	0.31	4.13	0.001
BIPQ personal control	1.68	0.41	0.33	4.06	0.001
HAD anxiety	0.84	0.17	0.40	5.03	0.001

## Discussion

This study investigated the impact of age, BMI, and psychological aspects on ED psychopathology among adults with T1D. Main findings include: (i) psychological aspects were associated with ED psychopathology among both male and female adults with T1D, in particular illness perceptions; and (ii) gender differences in the *associations* between psychological correlates and ED psychopathology existed among adults with T1D, but to a lesser degree than previously reported among our adolescent sample with T1D (Wisting et al., 2015).

The illness perception *personal control* seems to be of particular importance and the variable most strongly associated with ED psychopathology in the regression model among both males and females, that is, the more negative perceptions of personal control over their T1D the higher degree of ED psychopathology. The association between illness perceptions and ED psychopathology is in line with the results from adolescent females with T1D (Wisting et al., 2015). Illness perceptions, personal control especially, are also found to be important for diabetes self-care and metabolic control among both adolescents and adults with T1D (Fortenberry et al., 2014; Mc Sharry et al., 2011; McGrady et al., 2014), suggesting that this may be a particularly important aspect to consider in T1D care. The control aspect has also been described in qualitative research of comorbid T1D and ED. Qualitative approaches may be helpful to enhance understanding by capturing individual experiences and perceptions more in depth. Powers et al (2016) conducted focus groups to explore themes relevant to ED development and maintenance, and emerging themes included difficulty with control/coping. Participants described that the ED was something they could control, whereas diabetes just happened to them and was out of their control. This may be comparable and a complement to our quantitative findings that more negative perceptions of personal control are relatively strongly associated with ED psychopathology. The ED was described as a means of regaining control and coping with life stressors.

Other, not diabetes-specific, variables from the regression model among both males and females revealed that higher BMI and higher levels of anxiety were associated with ED psychopathology. These results are in line with the general ED literature (Ro et al., 2012; Swinbourne and Touyz, 2007). Additionally, age was significantly associated with ED psychopathology among females in the current study, that is, lower age was associated with higher levels of ED psychopathology. This is also consistent with existing ED literature that ED are more prevalent among females in their late adolescence and early adulthood. In summary, based on this regression model, it appears that some of the predictors of ED psychopathology in the general population may also be relevant ED predictors among individuals with T1D. Along those lines, it has been suggested women with ED and T1D most likely share underlying biopsychosocial risks (Goebel-Fabbri, 2020), and extending a generic ED risk model with T1D-specific aspects has been proposed. For example, the dual pathway model, suggesting that thin-ideal internalization, body dissatisfaction, and negative affect leads to ED psychopathology, (Stice et al., 2017) has been adapted to T1D (Rancourt et al., 2019). Furthermore, a T1D-specific ED risk model has been proposed, including meal planning to facilitate blood glucose control, higher BMI, depression and negative feelings, as well as intentional insulin omission (Goebel-Fabbri, 2009). This is partly in line with our findings regarding BMI and anxiety, although our study did not aim to test specific risk models systematically. Following the distinction of generic versus diabetes-specific aspects, we cannot rule out that our finding that anxiety was significantly associated with ED psychopathology may be owing to T1D-specific aspects such as fear of complications or fear of hypoglycemia rather than anxiety in general. Similarly, a distinction has been made that depression and diabetes-specific distress may constitute two qualitatively distinct concepts (Snoek et al., 2015). Along those lines, depression and anxiety as measured by the HADS in the current study may not be representative to individuals without diabetes.

### Gender and age differences

The overall gender differences demonstrated in the present study is generally in conjunction with previous studies. For example, although it is vital to acknowledge that also males suffer from ED, there is a consistently reported gender difference in the prevalence and presentation of ED. Variation in gender distribution exists across different ED diagnoses, but a general estimate of the male-to-female ratio of ED is 1–10 (Jacobi et al., 2004; American Psychiatric Association, 2013). Furthermore, in addition to gender differences in mean scores and prevalence rates, our findings regarding gender differences in the *associations* between psychological correlates and ED psychopathology somewhat correspond to our earlier research among adolescents with T1D (Wisting et al., 2015), though not as unequivocal as with adolescents. Based on these studies, it appears that gender differences in associations between potential psychological ED risk factors may diminish with age. Interestingly, a recent study (Smith et al., 2020) did not find significant sex differences in the associations between proposed ED risk factors in the modified dual pathway model (dietary regimen, BMI change, hunger/satiety disruption, dietary restraint, diabetes-specific negative affect, and body dissatisfaction) and ED psychopathology among adolescents and young adults with T1D. This might relate to the different correlates investigated, but more research is needed to investigate this further.

### Strengths and limitations

Although this study is strengthened by a wide age range and inclusion of males, there are also important limitations to be noted. First, this is a self-report, cross-sectional study and we can therefore not infer causality or directionality of our findings. For example, we cannot establish whether more negative perceptions of personal control leads to higher levels of ED psychopathology or the other way around. Also, data was collected from one diabetes outpatient clinic only, and we cannot be certain that this sample is representative for the T1D population in general. Furthermore, a generic measure of coping was used, which may be viewed as a weakness given that coping strategies have been found to depend on the particular situation and characterization of the challenge at hand (Hagger and Orbell, 2003; Seiffge-Krenke et al., 2009). This may also apply to our generic measure of depression and anxiety rather than a diabetes-specific measure of distress, such the Problem Areas of Diabetes (PAID) (Polonsky et al., 1995).

### Clinical and research implications

Broadly, the results of this study support the clinical relevance of health psychology, that is, the interplay between psychological and physical health. Acknowledging psychological aspects in a somatic clinical setting can enhance a more holistic treatment of chronic illness and potentially contribute to improved physical outcomes. Further, illness perceptions generally, and personal control specifically, was relatively strongly associated with ED psychopathology among both males and females. As mentioned above, this is also the case in the context of blood glucose control, suggesting the potential value of increased clinical awareness around such illness cognitions. For example, clinicians may want to consider using the Brief Illness Perception Questionnaire or similar questions as a starting point to examine individual perceptions or beliefs about their illness, including exploring with the patients whether they personally feel they can control their diabetes, the degree to which having diabetes affects them emotionally, how concerned they are about having diabetes, etc. Based on empirical findings, specific focus could be to promote patients’ sense of personal control over their T1D. Results regarding gender differences may also contribute to inform clinical work, as males and females might benefit from different approaches. Our studies of adolescents and adults suggest that psychological aspects may be important clinical targets among both adolescent and adult females, however be relevant among males at a later stage, that is, after having transitioned into adulthood. Intervention studies are needed to explore this further empirically. Finally, more qualitative or mixed method research would broaden our understanding of this complex comorbidity by complementing group level quantitative research with more in-depth individual perspectives and experiences.

## Conclusion

Clinical awareness of psychological aspects, in addition to age and BMI, may enhance detection of individuals at risk of developing ED, and potentially contribute to decrease the ED risk among this at-risk group. In particular, clinicians may want to consider means of promoting personal control among their patients with T1D.
